# RECRUITMENT LESSONS LEARNED FROM A RANDOMIZED PSYCHOEDUCATIONAL TRIAL FOR DEMENTIA FAMILY CAREGIVERS

**DOI:** 10.1093/geroni/igad104.3557

**Published:** 2023-12-21

**Authors:** Maria Aranda

**Affiliations:** University of Southern California, Los Angeles, California, United States

## Abstract

Family caregivers/care partners are the backbone of long-term services and support on behalf of persons living with memory loss such as Alzheimer’s and related dementias. Although there has been increased attention on family caregivers overall, documented recruitment strategies to participate in research—and clinical trials in particular—are lacking. This paper addresses the individual and system-level factors addressed in order to reach target recruitment goals in a community-based dementia caregiver trial conducted online with a racially- and ethnically-diverse cohort of mostly spousal/partner and adult children caregivers. Based on a sample of 231 trial participants and 105 focus group interviewees, we drew from our theoretical model—the Micro-Meso-Macro Framework—to elucidate factors that facilitated or impeded diverse cohort recruitment. We analyzed data from two data sources: a) source of referral and reason for contacting the recruitment team; and b) content analysis from focus group interviews. Based on our multi-level analysis we documented the following factors that facilitated trial participation: 1) MICRO: prior encounters with the research enterprise; educational attainment and occupational status; sense of altruism and beneficence; monetary incentivization; comfort with disclosure of private matters; starting from ground zero; and addressing gender issues; and 2) MESO/MACRO: assembling a proactive scientific team; leveraging logos and reputation; training trial staff and community partners; writing project budgets that incentivize diverse trial recruitment; making inroads with community partners; navigating technological challenges; and tapping into resources to alleviate barriers. In our paper, we will discuss lessons learned, and address future research directions including recruiting underrepresented groups.

